# Effect of Electrical Discharge Machining on Stress Concentration in Titanium Alloy Holes

**DOI:** 10.3390/ma9120957

**Published:** 2016-11-24

**Authors:** Wei-Hsuan Hsu, Wan-Ting Chien

**Affiliations:** Department of Mechanical Engineering, National United University, Miaoli 36063, Taiwan; u9911002@smail.nuu.edu.tw

**Keywords:** titanium alloy, Ti-6Al-4V, finite element method, electrical discharge machining, stress concentration

## Abstract

Titanium alloys have several advantages, such as a high strength-to-weight ratio. However, the machinability of titanium alloys is not as good as its mechanical properties. Many machining processes have been used to fabricate titanium alloys. Among these machining processes, electrical discharge machining (EDM) has the advantage of processing efficiency. EDM is based on thermoelectric energy between a workpiece and an electrode. A pulse discharge occurs in a small gap between the workpiece and electrode. Then, the material from the workpiece is removed through melting and vaporization. However, defects such as cracks and notches are often detected at the boundary of holes fabricated using EDM and the irregular profile of EDM holes reduces product quality. In this study, an innovative method was proposed to estimate the effect of EDM parameters on the surface quality of the holes. The method combining the finite element method and image processing can rapidly evaluate the stress concentration factor of a workpiece. The stress concentration factor was assumed as an index of EDM process performance for estimating the surface quality of EDM holes. In EDM manufacturing processes, Ti-6Al-4V was used as an experimental material and, as process parameters, pulse current and pulse on-time were taken into account. The results showed that finite element simulations can effectively analyze stress concentration in EDM holes. Using high energy during EDM leads to poor hole quality, and the stress concentration factor of a workpiece is correlated to hole quality. The maximum stress concentration factor for an EDM hole was more than four times that for the same diameter of the undamaged hole.

## 1. Introduction

Titanium alloys have several advantages, such as a high strength-to-weight ratio, favorable anticorrosion characteristics, and excellent biocompatibility [1]. Therefore, titanium alloys have been widely used in the construction of modern aircraft, automobiles, motorcycles, bicycles, sporting goods, and biomedical products [2,3,4,5]. Product design and manufacturing require holes to be machined on titanium alloys. Hole quality is a critical factor for product performance and reliability. Thus, the machinability of titanium alloys has been extensively studied for avoiding burr and distortion of machined holes and enhancing processing quality.

In 2005, Che-Haron and Jawaid confirmed that titanium alloys are difficult to machine because of their low thermal conductivity and high chemical reactivity with cutting-tool materials [6]. Therefore, when conventional cutting methods are employed to machine titanium alloys, temperature increase is inevitable and the resulting high temperatures lead to galling and welding between the tool and workpiece. This phenomenon not only leads to rapid tool wear and failure but also causes surface damage to titanium alloy workpieces. In the same year, Cantero et al. [7] conducted research on the dry drilling of a titanium alloy, Ti-6Al-4V. They studied tool wear by using optical microscopy and scanning electron microscopy with energy-dispersive X-ray spectroscopy (SEM–EDS). Additionally, they estimated the quality of machined holes in terms of geometrical accuracy and burr formation. Ti-6Al-4V is a difficult-to-machine material and its machinability is poor with conventional cutting methods. Therefore, using conventional cutting methods, the applications of Ti-6Al-4V are limited [8]. Recently, to improve the processing quality of titanium alloy, the milling parameters of Ti-6Al-4V were analyzed through an image processing method and the results confirmed that Ti-6Al-4V titanium alloy milling induces a high content of β phase and small grain size on the machined surface. This research indicates the effect of milling parameters on a machined surface and is expected to improve Ti-6Al-4V machinability [9].

Electrical discharge machining (EDM) is a nontraditional machining process. EDM is based on thermoelectric energy between a workpiece and an electrode. During EDM, a pulse discharge occurs in a small gap between the workpiece and electrode. Then, the material from the workpiece is removed through melting and dispersal. EDM is suitable for machining materials that are extremely hard, brittle, wear resistant and electrically conductive [10]. In comparison with conventional mechanical methods for machining titanium-related materials, EDM can effectively reduce tool cost and improve product quality [11,12]. In 2007, different electrode materials, such as graphite, copper, and aluminum were used to investigate the influence of processing parameters, such as pulse current and pulse-on duration on the machinability characteristics of Ti-6Al-4V [13]. The results showed that an increase in pulse current or pulse-on duration enhanced the material removal rate, surface roughness, electrode wear and recast layer thickness. When EDM is used to machine a hole, a recast layer is formed around the boundary of the EDM hole and defects such as cracks and notches are often detected at the hole boundary. The recast layer and microcracks adversely affect EDM hole quality [14]. Although using EDM to machine Ti-6Al-4V is feasible, the recast layer thickness is increased with an increase in the peak current or pulse-on time [15]. EDM changes the material properties of Ti-6Al-4V; specifically, the surface roughness of the alloy is increased. The degree of severity of defects caused by EDM is proportional to the applied peak current [16]. The quality of an EDM-modified surface is influenced by the discharge energy magnitude. Lower electrical discharge energy results in a better machined surface. A large pulse current and long pulse-on duration in EDM damage the machined surface, thereby reducing material strength. Such damage deteriorates the integrity of the machined surface and causes stress concentration. In addition to the quality of the machined surface, the material removal rate (MRR) and tool wear ratio are also assumed as a performance indexes of EDM process. Previous studies confirm that MRR is strongly influenced by peak current, pulse on time and spindle rotational speed [17]. An increase in these parameters results in an increase in MRR. Additionally, the electrode wear is directly proportional to the energy density. 

The degree of stress concentration is affected by geometrical parameters, such as cavity shape, hole shape and corner radius. However, generally speaking, for easy manufacturing and assembly, designing members with holes is inevitable. Thus, stress concentration in a member is difficult to avoid. Stress concentration may cause cracks around a hole and adversely affect the product life cycle. Therefore, because of its importance in the product life cycle, stress concentration in holes has been investigated in detail [18,19]. However, the measurement of stress concentration in a member with a hole under various loads is a difficult and tedious task. To improve research efficiency, previous investigators have used finite element simulations to study stress concentration. Kubair and Bhanu-Chandar employed the finite element method to investigate stress concentration factors in functionally graded panels with a circular hole under uniaxial tension [20]. Moreover, Yang et al. confirmed that structural cracks typically start from the stress concentration position [21]. Stress concentration can severely affect the structural strength of engineering components. Therefore, studying the stress concentration factor is crucial for the fracture life assessment of such components. The stress concentration factors of various geometries have been investigated, with results presented in the form of charts [22]. In addition, stress concentration factors under different loading conditions have been reported [23]. However, the effects of structural defects on the stress concentration are not yet taken into account. The influence of processing damage on the stress concentration factor is complex. Obtaining an analytical solution for the stress concentration factor of a member with arbitrary damage is difficult. Therefore, damage or defects are generally ignored in stress concentration investigation.

Titanium alloy plates with holes are used in different fields for various purposes. The quality of holes affects stress distribution in a component and poor hole quality may result in structural damage or crack propagation. Furthermore, the quality of an EDM hole greatly depends on EDM parameters. The influence of structural defects on the stress concentration factor is not mentioned in design handbooks. Research on stress concentration in EDM specimens is still incomplete. For ensuring the optimal mechanical performance and durability of titanium alloy products, understanding the characteristics of EDM holes is essential. In this study, the finite element method (FEM) was employed to evaluate the effects of EDM parameters on the stress concentration factor of EDM holes and to identify optimal EDM parameters for the hole-machining surface quality.

## 2. Experimental Procedure

### 2.1. Materials

Ti-6Al-4V used in this investigation was a 21-mm-thick hot-rolled plate with a chemical composition (wt %) of 6.0–6.1 Al, 4.1–4.2 V, 0.13–0.16 Fe, 0.02–0.026 C, 0.02 N, 0.003 H, and 0.10–0.11 O, with Ti constituting the remainder. Table 1 lists the physical properties of this alloy.

### 2.2. Experimental Setup

**Figure 1:**
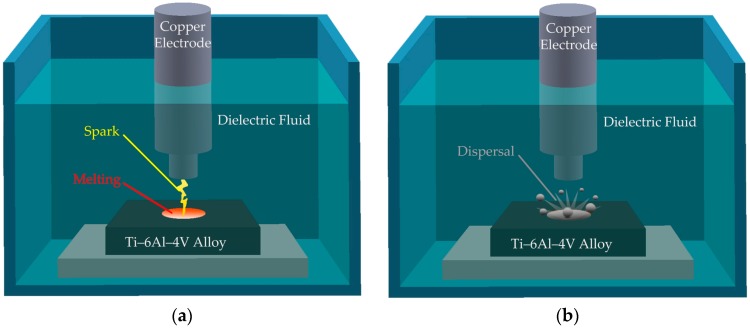
Schematic drawing of EDM process: (**a**) melting and (**b**) dispersal.

An electrical discharge machine (Charme CD-50 M, Ching Hung Machinery & Electric Industrial Co., Ltd., Taichung, Taiwan) with servo control was used to conduct the experiments. In general, the profile of the hole is affected by the electrode material and discharge energy used in the EDM process. In the regard of the electrode material, a tungsten carbide electrode permits the obtainment of a more regular top profile of the hole than the copper-based electrodes [24]. However, a copper electrode has been widely used in the industry because it has the advantage of low cost and high stability. Thus, a copper electrode is used in the experiment. Besides, according to previous study results [17], discharge energy, based on peak current, voltage and frequency, has an influence on the surface quality of the hole. Among these parameters, peak current is one of the main parameters affecting the surface quality of the hole. In this study, the aim is to develop a method for rapidly evaluating the stress concentration factor of EDM holes. In order to clearly present the results, the pulse current is selected to present the results. The EDM processing parameters were as follows: processing depth, 0.5 mm; voltage, 25 V; pulse current, 2 A–3 A; pulse-on duration, 100 μs; pulse-off duration, 20 μs. Figure 1 schematically displays the EDM process; a copper electrode with a diameter of 1 mm had the positive polarity and the Ti-6Al-4V alloy had the negative polarity during the process. Commercial-grade kerosene was used as the dielectric fluid in all experiments. 

### 2.3. Data Analysis Instrument and Method

SEM (JSM 6500-F, JEOL, Tokyo, Japan) was employed to obtain high-optical-contrast microscale images of EDM holes for measuring their profiles. After obtaining SEM micrographs, imaging software (Image-Pro Express) was employed to analyze the holes and calculate the coordinates of hole contours. Transverse profiles of the holes were then used to analyze the degree of stress concentration in the holes.

**Figure 2:**
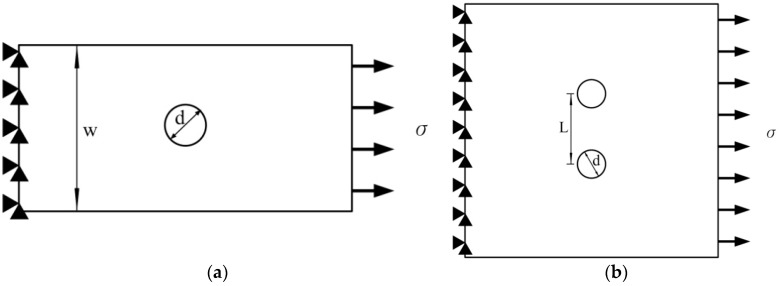

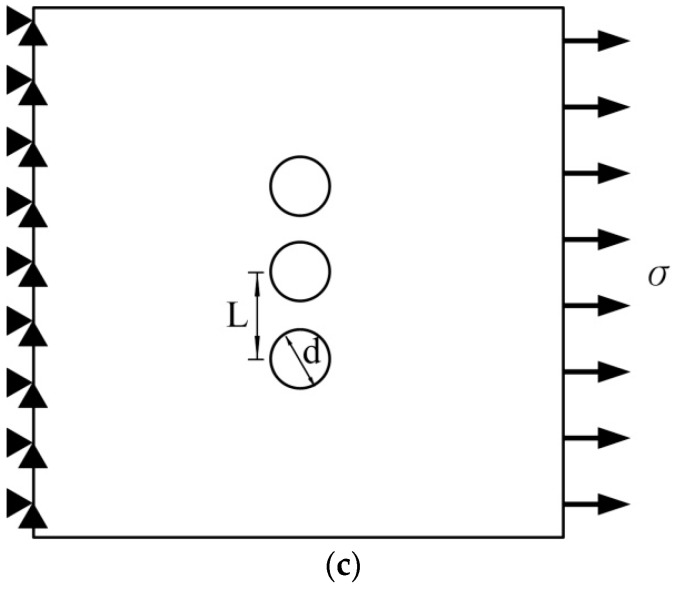
Schematic diagrams of geometries used in simulations: (**a**) specimen with single hole; (**b**) specimen with two holes and (**c**) specimen with three holes.

In this study, the stress distribution was evaluated through FEM. In FEM simulations, a commercial program (COMSOL Multiphysics) was employed to evaluate the stress characteristics of holes machined through EDM in a titanium alloy. Figure 2 shows the shape of the computational domain and dimensions of the EDM specimen, in which w, d, and L represent the width of the specimen, hole diameter and distance between adjacent holes, respectively. The lateral dimension of the specimen was considerably larger than its thickness. Thus, the plane stress condition was used in the finite element model. The specimen was fixed at the left edge (implying that displacements in the x and y directions were zero at the left edge), and uniform pressure (σ) was applied at the right edge of the specimen. The relationship between the hole diameter and specimen geometry is described as follows. For a specimen with a single hole (Figure 2a), the d/H ratio was 0.1–0.5. For a specimen with two holes (Figure 2b), the L/d ratio was 3–7. For a specimen with three holes (Figure 2c), the d/L ratio was 0.3–0.6. Moreover, the transverse profiles of holes obtained from image processing were incorporated into the FEM model to identify stress concentration in the holes under various EDM conditions.

### 2.4. Prediction of Stress Concentration Factor

Most mechanical parts contain complex features such as grooves, holes, notches, and screw threads. These complex geometries cause nonuniform stress distribution; moreover, rapid changes in the structure of a section cause an increase in stress. The localization of the aforementioned high stresses is called stress concentration. Moreover, the ratio of the nominal stress and maximum stress is called the stress concentration factor, which can be used to estimate the extent of stress concentration. The stress concentration factor of mechanical parts can be expressed as Equation (1) [23,25]:

(1) $$K=\frac{\sigma_{\max}}{\sigma_{n}}$$

where σ_max_ and σ_n_ are the maximum stress and nominal stress, respectively.

In the analysis of the stress concentration factor, the maximum stress was determined using the FEM analysis results. The nominal stress acting on a net cross-sectional area under axial loading was defined by Equations (2) and (3) for specimens with a single hole and plural holes, respectively.

(2) $$\sigma_{n}=\frac{\sigma }{\left(1-\frac{d}{W}\right)}$$

where σ represents the applied pressure at the right edge of the specimen.

(3) $$\sigma_{n}=\frac{\sigma }{\left(1-\frac{d}{L}\right)}$$

The conventional empirical formulas for the stress concentration factors of a finite-width thin specimen under tension with one, two and three circular holes are given by Equations (4)–(6), respectively. These three empirical formulas were proposed by Howland, Haddon, and Schulz, respectively, and are satisfactory for numerous design applications when structural defects caused by holes are ignored.

(4) $$K=2+0.284\left(1-\frac{d}{W}\right)-0.6{\left(1-\frac{d}{W}\right)}^{2}+1.32{\left(1-\frac{d}{W}\right)}^{3}$$

(5) $$K=3.003-3.126\left(\frac{d}{L}\right)+0.4621{\left(\frac{d}{L}\right)}^{2}$$

(6) $$K=3-3.095\left(\frac{d}{L}\right)+0.309{\left(\frac{d}{L}\right)}^{2}+0.786{\left(\frac{d}{L}\right)}^{3}$$

## 3. Results and Discussion

### 3.1. EDM Results and Stress Distribution

**Figure 3:**
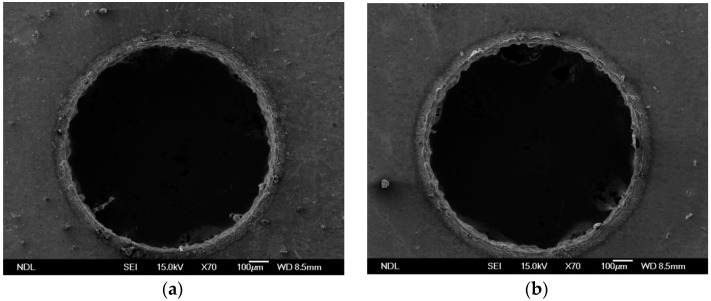
SEM image of EDM holes machined at applied pulse current of (**a**) 2 A and (**b**) 3 A.

**Figure 4:**
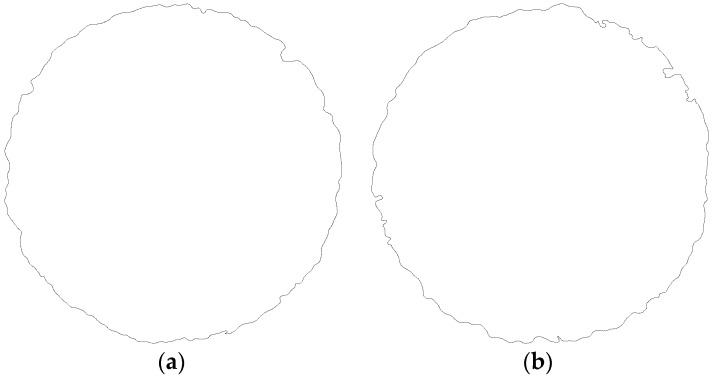
Results of processing images in Figure 3: (**a**) hole profile obtained at 2 A pulse current and (**b**) hole profile obtained at 3 A pulse current.

EDM holes on Ti-6Al-4V obtained using a pulse current of 2 A and 3 A are shown in Figure 3a,b respectively. The SEM images were converted into black-and-white binary images for convenience in FEM analysis. Results of the processing of SEM images in Figure 3a,b are shown in Figure 4a,b, respectively. The transverse profile of an EDM hole was an irregular hole edge. The resulted processing image revealed that the quality of EDM holes was directly related to the strength of the applied current. When the pulse time was fixed, an increase in the pulse current deteriorated the contour quality of EDM holes.

**Figure 5:**
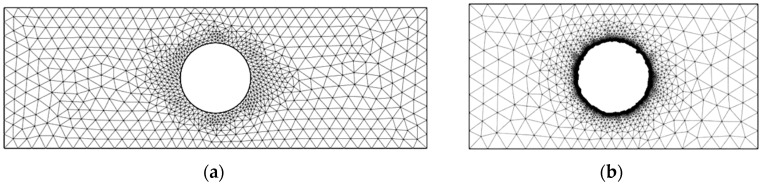
FEM meshes for titanium alloy specimen with single hole: (**a**) ideal hole and (**b**) EDM hole obtained using 2 A pulse current.

**Figure 6:**
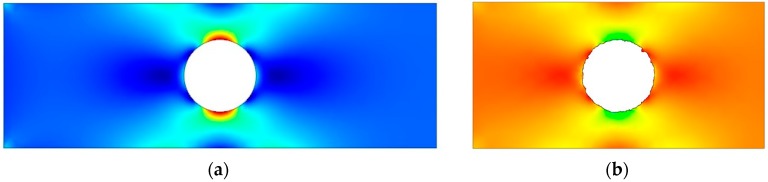
FEM calculation results for stress distribution: (**a**) stress contour of ideal hole and (**b**) stress contour of EDM hole obtained using 2 A pulse current.

To evaluate the stress concentration in EDM holes in the titanium alloy, the transverse profiles of the holes were input to the FEM software to create an FEM model and subsequently determine the stress field and stress concentration factor. Figure 5 shows the FEM mesh model for the specimen with a single hole. Meshes for an ideal (undamaged) hole and an EDM hole are shown in Figure 5a,b, respectively. A local fine mesh along the hole edge was used in the FEM model. Figure 6 shows the analysis results for the case in Figure 5. Figure 6a, showing results for an ideal hole, reveals nonuniform stress distribution across the section through the center of the hole. In the specimen with the ideal hole, the maximum stress appeared at the minimum cross section of the specimen. Figure 6b shows the stress states in the titanium alloy with an EDM hole. This figure clearly indicates that the maximum stress occurred at the defect; this changed the hole profile and altered the stress distribution around the hole. Furthermore, the stress distribution in the specimen with multiple holes was similar to that in the specimen with a single hole. In specimens with ideal holes, the maximum stress always appeared at the minimum cross section of the specimen. When the ideal hole profiles were replaced with EDM hole profiles, the maximum stress occurred at the defect near the minimum cross section of the specimens.

### 3.2. Stress Concentration Factor of Specimens with EDM Holes

**Figure 7:**
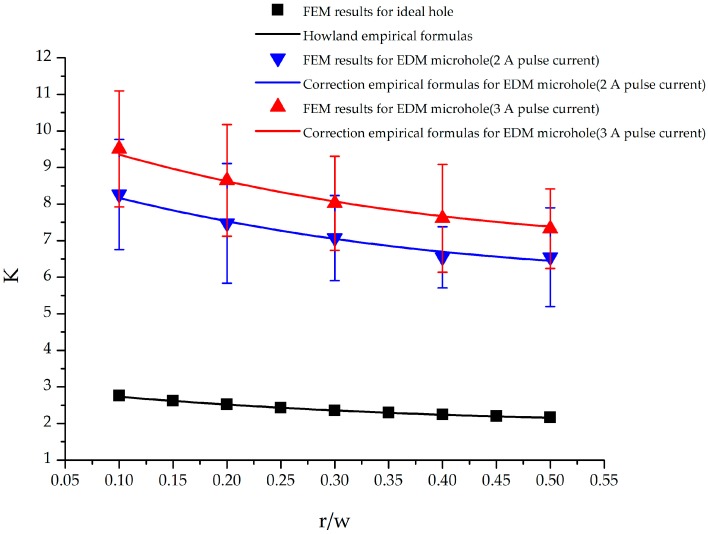
Stress concentration factors of a titanium alloy specimen with a single circular hole under external axial load.

Figure 7 shows the stress concentration factors of a titanium alloy specimen with a single circular hole under an external axial load. The analysis cases included specimens with an ideal hole and EDM hole. In addition, results predicted using the empirical formula of Equation (4) are also included in Figure 7. All cases in Figure 7 show that an increase in the r/w ratio resulted in a decrease in the stress concentration factor. For the specimen with an ideal hole, the FEM results showed good agreement with the results calculated using Equation (4). The average difference between the stress concentration factors obtained using the FEM results and empirical formula was approximately 0.27%. Moreover, the stress concentration factor for the EDM hole was greater than that for the ideal hole. For the ideal hole, stress concentration factors of 2.6 and 2.17 were observed at r/w values of 0.1 and 0.5, respectively. For the EDM hole fabricated using a pulse current of 2 A, the stress concentration factors were 8.26 and 6.55 at r/w values of 0.1 and 0.5, respectively. These stress concentration factor values were approximately 3.18 and 3.02 times the corresponding values for the ideal hole. Moreover, when the pulse current was increased from 2 A to 3 A, the stress concentration factors also increased to 9.51 and 7.32 for r/w values of 0.1 and 0.5, respectively. These values were approximately 3.66 and 3.38 times the corresponding values for the ideal hole.

**Figure 8:**
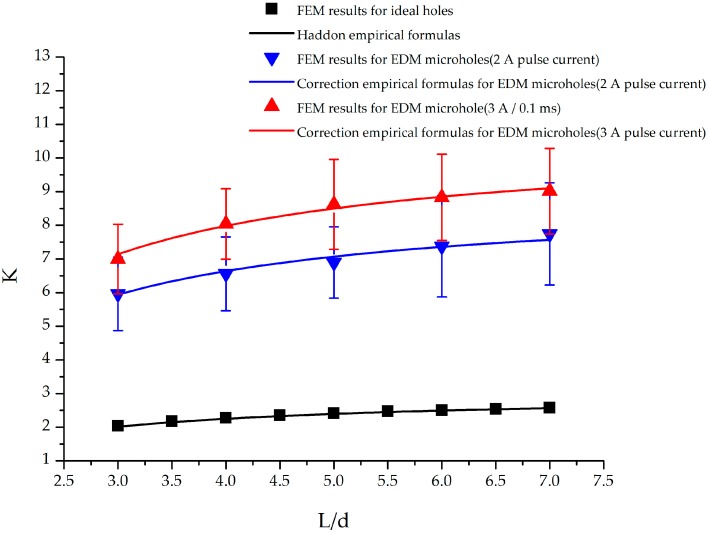
Stress concentration factors of a titanium alloy specimen with two circular holes under external axial load.

The stress concentration factors of a specimen with two circular holes under an external axial load are shown in Figure 8. The stress concentration factor of this specimen was dependent on the L/d ratio: an increase in L/d resulted in an increase in stress concentration factor. To compare the calculation results obtained using the empirical formula with the FEM analysis results for the specimen with two ideal holes, stress concentration factors calculated using the formula in Equation (5) were compared with those obtained from the FEM analysis results. When the L/d ratio varied from 3 to 7, the average difference between the stress concentration factors obtained using the FEM simulation results and empirical formula was approximately 0.9%.

For the EDM hole fabricated using a pulse current of 2 A, the stress concentration factors were 5.96 and 7.74 at L/d values of 3 and 7, respectively. These stress concentration factor values were approximately 3.3 times the corresponding values for the ideal hole. For the EDM hole fabricated using a pulse current of 3 A, a stress concentration factor of 7 was observed at an L/d value of 3. Moreover, when L/d was increased to 7, the stress concentration factor increased to 9.02. The stress concentration factor values for EDM holes fabricated at a 3 A pulse current were more than 3.8 times those for ideal holes.

**Figure 9:**
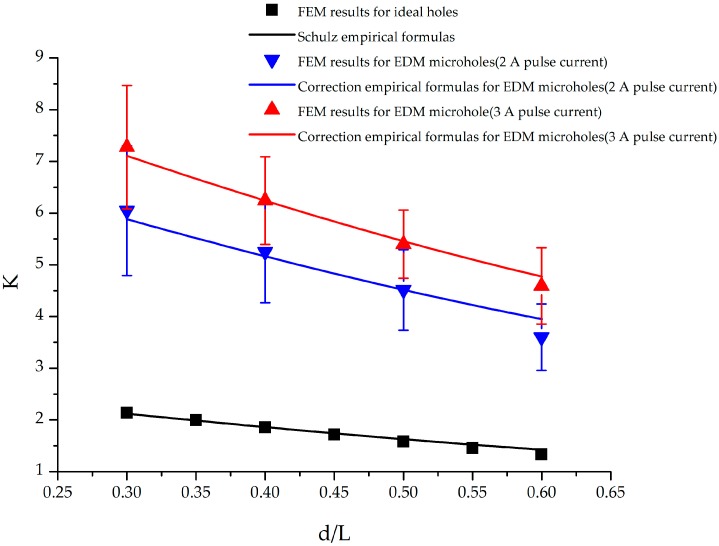
Stress concentration factors of a titanium alloy specimen with three circular holes under external axial load.

Figure 9 shows the relationship between the stress concentration factors and d/L ratio for a specimen with three circular holes. All cases indicate that an increase in d/L resulted in a decrease in the stress concentration factor. In the case of ideal holes, stress concentration factor values calculated using the empirical formula agreed well with those obtained using FEM calculations. Furthermore, when d/L varied from 0.3 to 0.5, the difference between the stress concentration factors obtained using the FEM calculations and empirical formula was less than 1.14%. When d/L was increased to 0.6, this difference increased to approximately 6.29%. The average difference between the two stress concentration values for d/L varying from 0.3 to 0.6 was approximately 2.34%.

Figure 9 shows that the stress concentration factors for EDM holes were greater than those for ideal holes. For ideal holes, stress concentration factors of 1.79 and 1.11 were observed at d/L values of 0.3 and 0.6, respectively. For EDM holes fabricated using a 2 A pulse current, the stress concentration factors were 6.05 and 3.6 for d/L values of 0.3 and 0.5, respectively. These stress concentration factor values were approximately 3.38 and 3.24 times the corresponding values for ideal holes. When the pulse current was further increased to 3 A, the stress concentration factor values for EDM holes became more than four times the corresponding values for ideal holes. On the basis of these findings, we can conclude that EDM parameters have a substantial influence on the machining surface profile, and the stress concentration factor of a specimen is directly correlated with the quality of the EDM hole profile.

### 3.3. Proposed Empirical Formulas for Stress Concentration Factor of Specimen with EDM Holes

On the basis of the findings listed in Section 3.2, an empirical formula for calculating the stress concentration factors of Ti-6Al-4V titanium alloy specimens with EDM holes was constructed. The correction empirical formula in Equation (7) contains two items. The first item K is the stress concentration factor for ideal holes obtained from the conventional empirical formulas given by Equations (4)–(6). The second item Cn is a proposed correction term. Equations (8)–(10) represent the proposed correction term for specimens with one, two, and three EDM holes, respectively.

(7) $$K_{\mathrm{EDM}}=K\times C_{n}$$

where the subscript n in C_n_ denotes the number of holes in the specimen.

(8) $$C_{1}=-0.187A^{2}+1.369A+1$$

where A represents the applied pulse current in EDM.

(9) $$C_{2}=-0.126A^{2}+1.227A+1$$

(10) $$C_{3}=-0.103A^{2}+1.093A+1$$

During fitting, the obtained coefficient of determination, R^2^, was 0.735, 0.776 and 0.8 for specimens with one, two, and three EDM holes, respectively. The empirical formulas provided a suitable description of the relationship between the stress concentration factor and EDM pulse current. To verify the validity of the correlation empirical formula, stress concentration factor values calculated using the correlation empirical formula were compared with those obtained using the FEM analysis results. The average difference between the two was 1.01%, 1.2%, and 2.7% for specimens with one, two, and three EDM holes, respectively. Therefore, values calculated using the proposed formulas were in good agreement with the FEM results, and the formulas can be used to determine the appropriate EDM pulse current for different applications.

## 4. Conclusions

EDM is an excellent hole fabrication technique because of high machining efficiency and the capability of machining all conductive materials regardless of their hardness. However, EDM has the disadvantage of relatively low machining quality. Thus, an innovative method was proposed for estimating the quality of EDM holes. The method combining FEM and image processing can rapidly evaluate the stress concentration factor of a workpiece. To prove the correctness of this method of evaluation in stress measurement, FEM results were compared with the empirical formulas in the same study case. The FEM results showed good agreement with the results calculated using the empirical formulas. In experiment, Ti-6Al-4V was used as an experimental material due to its excellent mechanical properties and applicability. According to experimental results, EDM holes have irregular profiles and a higher stress concentration factor when using a higher pulse current. When a 2.0 A pulse current was used for EDM, the stress concentration factor for EDM holes was approximately 3.02–3.18, 3.26–3.34, and 3.24–3.38 times that for undamaged holes in specimens with one, two, and three holes, respectively. When the pulse current was increased to 3 A, the stress concentration factor for EDM holes increased to approximately 3.38–3.66, 3.82–3.89, and 4.06–4.13 times that for undamaged holes in specimens with one, two, and three holes, respectively. Poor EDM hole surfaces were found to produce large stress concentration factors, and correlation empirical formulas were proposed to determine the processing quality of EDM holes on the Ti-6Al-4V titanium alloy. The stress concentration factor calculated using the correlation empirical formulas can be used as an index to evaluate the average gap between the ideal profile and the real profile. Analytical and experimental results confirmed that the proposed method can be used to estimate the stress concentration factor of EDM specimens. Furthermore, FEM can be employed to improve EDM performance.

## Figures and Tables

**Table 1:** Physical properties of Ti-6Al-4V.

Property	Value
Density	5010 kg/m^3^
Young’s modulus	110.3 GPa
Poisson’s ratio	0.33
Yield strength	1.57 GPa
Thermal conductivity	7 W/(m·K)
Electrical conductivity	1.01% IACS
